# Evaluation of a structured individualised protocol as a potential cost‐effective diagnostic and therapeutic approach to chronic diarrhoea in the dog

**DOI:** 10.1002/vms3.154

**Published:** 2019-02-11

**Authors:** Christine E. Bryan, Jeb C. Cade, Andrew J. Mackin, Alyssa M. Sullivant

**Affiliations:** ^1^ Department of Clinical Sciences Mississippi State University College of Veterinary Medicine Mississippi State Mississippi USA

**Keywords:** diarrhoea, large bowel, small bowel

## Abstract

Diagnostic investigation and management of chronic diarrhoea in dogs can be cost‐prohibitive to many owners. The objectives of this study were to evaluate evidence‐based, individualised diagnostic and therapeutic protocols for management of dogs with chronic diarrhoea, where financial constraints dictate a budget‐limited approach and where more expensive approaches are deferred until simple affordable protocols are unsuccessful. Twenty‐two client‐owned dogs with chronic (minimum 2 weeks duration) untreated small, large or mixed small/large bowel diarrhoea were enrolled in a budget‐limited step‐wise management protocol (maximum expenditure $300 over 6 weeks), with diagnostic testing and therapeutic trials managed in an individualised and evidence‐based fashion. Success was defined as complete resolution of diarrhoea for a minimum of 1 month. Dogs that failed to respond to a budget‐limited protocol were then enrolled for complete, referral‐level management. Four dogs exited the project early (one death due to caval syndrome, three lost to follow‐up). Thirteen out of the remaining 18 dogs had complete resolution of diarrhoea utilising a budget‐limited approach (success rate 72.2%, confidence intervals 46.5–90.3%) and five dogs were moved on to a referral‐level investigation, with complete resolution of diarrhoea in four out of five. Seventeen out of the 18 dogs therefore responded to a protocol based on a budget‐limited approach followed by extensive investigation only if needed, for an overall success rate of 94.4% (CI 72.7–99.9%). Comprehensive investigation of chronic diarrhoea can be deferred while simple affordable diagnostics and therapeutic trials are conducted in stable canine patients and, often, an extensive management approach will be unnecessary.

## Introduction

Many cases of diarrhoea in dogs are mild and self‐limiting. Unfortunately, however, some dogs develop chronic, persistent diarrhoea that can be highly distressing to both pet and owner. Both small bowel and large bowel diarrhoea can have a significant impact on the quality of life of the affected dog and, in advanced cases, can even be life‐threatening.

Veterinarians typically find chronic diarrhoea in dogs to be challenging. In many cases, referral to a veterinary specialist‐level facility is suggested for advanced diagnostic procedures such as abdominal ultrasonography, gastrointestinal endoscopy and/or laparoscopy. Unfortunately, referral‐level diagnostic and therapeutic management of chronic diarrhoea can be beyond the financial resources of many dog owners.

The Mississippi State University College of Veterinary Medicine (MSU‐CVM) primary care (non‐referral) service clinicians have frequently encountered local dogs with chronic diarrhoea whose owners could not afford referral‐level investigation. The MSU‐CVM small animal internal medicine referral service clinicians have also frequently provided advice to area practitioners dealing with dogs with chronic diarrhoea whose owners could not afford referral. Based on this experience, both groups of clinicians have made the same observation: with diagnostic and therapeutic management carefully tailored to be affordable, many cases of chronic diarrhoea in dogs can be effectively resolved without resorting to more expensive referral‐level strategies.

We hypothesised, based on our experience, that many dogs (about 50% or more) with chronic diarrhoea could be successfully managed in general practice using evidence‐based and individualised diagnostic and therapeutic protocols that are affordable to most owners, without the need for high cost referral‐level procedures such as abdominal ultrasound and endoscopy. We also hypothesised that, if a full referral‐level investigation was instituted only in those dogs that failed to responded to a structured step‐wise approach utilising affordable protocols, eventual success rates for resolution of diarrhoea would be significantly >50%. The primary purpose of this current study was to develop and evaluate the success of an evidence‐based and individualised approach to diagnostic and therapeutic protocols for use by general practitioners in dogs with chronic diarrhoea, in which financial constraints dictate a practical, affordable and budget‐restricted approach and in which more high cost management was deferred until simple affordable protocols had proven unsuccessful.

The establishment of a model for developing affordable protocols that can be proven to be reasonably effective compared to complete referral‐level management plans would help to bridge the divide between those pet owners who can afford extensive investigations and those who cannot afford specialist care.

## Materials and methods

### Phase One

The purpose of Phase One of our study was to determine the proportion of dogs with chronic small and/or large bowel diarrhoea that could be successfully treated using affordable and budget‐restricted diagnostic and therapeutic plans. Cases of canine chronic diarrhoea (the initial study goal was to recruit up to 50 cases in total if needed, over a period of up to 2 years) were recruited for the study through the canine patient population seen through the primary care practice at MSU‐CVM and through the local general practices in the Mississippi, Alabama and Tennessee region that were serviced by the MSU‐CVM small animal internal medicine referral service. Cases were recruited by advertisement efforts targeted at regional veterinarians (mailed informational flyers and presentations at regional veterinary meetings) and local pet owners (press releases and web‐based information made available through social media efforts).

Inclusion criteria for canine chronic diarrhoea cases included the following:

1. Diarrhoea of at least 2 weeks duration,
2. No veterinary diagnostic testing undertaken since the onset of diarrhoea, and
3. No veterinarian‐directed therapy administered for at least 3 months prior to the commencement of study.

Exclusion criteria included persistent anorexia and recent (within 3 months) administration of anthelmintics, antibiotics, gastrointestinal protectants, prokinetics, immunosuppressants and novel protein or hydrolysed diets.

A comprehensive diagnostic and therapeutic investigation, often including diagnostics such as complete blood count (CBC), serum biochemical panel, urinalysis, abdominal radiographs and ultrasound, adrenocorticotropic hormone stimulation testing and endoscopy, was always offered, as indicated, to veterinarians and pet owners that contacted the authors regarding the study. The costs of recommendations of this nature typically ranged from $300 to $3000. Enrolment in the study was limited to those pet owners who, typically for financial reasons, declined this comprehensive approach. Local dogs enrolled in the study were managed by two of the authors (CEB, JCC) within the MSU‐CVM primary care service. Dogs from outside the client base of the MSU‐CVM primary care service were managed by their local veterinarians, under the advice and guidance of the authors (CEB, JCC, AJM, AMS).

Comprehensive history and physical examination information, including faecal consistency scores (Greco D. Diagnosis and dietary management of gastrointestinal disease. Purina Veterinary Diets Quick Resource Plan, Société des Produits Nestlé S.A., Switzerland. Available at: https://www.purinaproplanvets.com/media/1202/gi_quick_reference_guide.pdf. Accessed 28 March 2018), was collated at the time of admission, either by the researchers or the collaborating local veterinarians. Individualised initial diagnostic and therapeutic plans were then devised by the four authors based on signalment, history and physical examination. Prior to the commencement of the study, the authors met on multiple occasions to develop and refine broad evidence‐based cost‐effective protocols for the management of chronic diarrhoea (both small and large bowel diarrhoea), although it was acknowledged that such protocols would be individualised and modified based on the specific circumstances of each patient. Large bowel diarrhoea was characterised by an increased frequency of defaecation, typically accompanied by excessive faecal mucus, haematochezia and tenesmus (Leib 2000; Lecoindre & Gaschen 2011), whereas small bowel diarrhoea was characterised by features such as anorexia, weight loss, melaena, increased faecal volume and watery faeces (Allenspach *et al*. 2007; Volkmann *et al*. 2017). At the time of enrolment of each new patient and weekly thereafter for the duration of the study, the authors would meet as a committee to discuss the ongoing management of individual cases, using evidence‐based source material when available (Leib 2000; Westermarck *et al*. 2005a,b; Allenspach *et al*. 2007; Fogle & Bissett 2007; Hall 2011; Kilpinen *et al*. 2011, 2014; Lecoindre & Gaschen 2011; Chaitman *et al*. 2016; Paap *et al*. 2016; Volkmann *et al*. 2017). The primary sources of evidence were articles derived from electronic searches using three medical, agricultural and/or biological databases: Medline, Agricola and CAB (Centre for Agriculture and Bioscience International) Abstracts. Key word searches were conducted prior to the study, in order to develop initial protocols and then as the need arose for individual cases during weekly group discussions. Relevant articles that were identified via an electronic search were obtained from on‐line resources or copied from journal hard copies in the veterinary teaching hospital libraries or from collaborating libraries.

Each enrolled case was provided with up to $300 (United States Dollars) for the complete diagnostic and therapeutic approach, an amount that was estimated to be affordable by most pet owners and still sufficient to fund reasonable management plans. For patients enrolled at the MSU‐CVM primary care clinic, standard fees were charged, but subsidised by the project up to a total funding of $300. For patients enrolled through their local veterinary practice, the practice was sent $300 for each enrolled case and advised to charge standard fees for services provided.

For each enrollee, diagnostic and therapeutic protocols were generated based on the history, physical examination and clinical signs of each patient, but often included inexpensive diagnostic tests such as faecal flotation and faecal cytology (and, less frequently, faecal culture and rectal scrapings) and therapeutic trials including food trials, parasiticides, antibiotic trials with drugs such as tylosin and metronidazole and probiotics. Initial diagnostic and therapeutic plans and long‐term weekly advice and follow‐up (minimum of 3 months), were provided by the authors to clients (cases seen at MSU‐CVM) or collaborating practitioners utilising established tools such as standardised faecal consistency scores. Management plans were reviewed weekly and altered as indicated (typically, a progressing series of simple diagnostic tests and therapeutic trials) until diarrhoea resolved.

Treatment success was defined by the primary end point of normal faecal consistency scores for at least 1 month following completion of the treatment period. Treatment failure was defined as persistent or recurrent diarrhoea despite 6 weeks of various cost‐effective treatment protocols or relapse of diarrhoea within a month of completion of the initial 6‐week management phase or patient ill‐health that was determined by the investigators to be severe enough to warrant early transition to Phase Two of the study.

### Phase Two

The purposes of Phase Two of our study were to determine the proportion of patients that had persistent diarrhoea despite an initial affordable budget‐restricted diagnostic and therapeutic approach and still required a more expensive, referral‐level management plan and to determine, if possible, the exact cause of diarrhoea in those patients that failed to respond to the initial budget‐restricted protocols.

Dogs enrolled in Phase One of the study that still had chronic diarrhoea at the end of the study period (defined as 1 month following completion of the prescribed treatment regimen), or that were determined to be too unstable to continue with the full Phase One study, were referred to the MSU‐CVM small animal internal medicine referral service, under the direct supervision of one of the two study investigators who was board‐certified in internal medicine (AJM, AMS). Individualised referral‐level diagnostic and therapeutic plans were devised by the investigators based on the specific case details. Each enrolled case was funded for the full amount needed for a full referral investigation and appropriate therapy, as determined by the investigators (estimated to be, typically, $1500–$3500). Patients were enrolled in Phase One of the study with the understanding that, if the diarrhoea failed to resolve, a full referral‐level investigation would be available at no cost to the owner.

Diagnostic and therapeutic plans were individualised based on the circumstances of each patient, but could reasonably be expected to include diagnostic tests such as a CBC, serum biochemical profile, abdominal radiography and ultrasonography, gastrointestinal panels (including folate and cobalamin levels) and gastrointestinal biopsy via endoscopy, laparoscopy or exploratory laparotomy and targeted and aggressive therapy based on a final definitive diagnosis (Allenspach *et al*. 2007). Long‐term follow‐up was then conducted as for Phase One.

### Protocol development

The purpose of the Protocol Development component of our study was to generate several usable, practical and evidence‐based affordable protocols available for dissemination to practitioners, supported by evidence generated during our study quantifying the likelihood of success associated with budget‐restricted diagnostic and therapeutic compromises. As part of the ‘art’ of veterinary medicine is tailoring diagnostics and therapy to the individual patient, the authors chose not to use predetermined, rigid protocols for managing the individual dogs enrolled in this study. Prior to the commencement of study and throughout the duration of the project, however, the investigators met regularly to develop and refine evidence‐based approaches for each issue that they encountered. By the time of completion of the study, it was anticipated that two protocols (one for small bowel diarrhoea and one for large bowel diarrhoea) would be developed to serve as broad templates from which individualised management approaches could be developed for specific patients.

### Animal use

An animal use proposal for the study was submitted to the Mississippi State University Institutional Animal Care and Use Committee. However, as all enrolled patients would receive only diagnostic and therapeutic interventions that were indicated and in best health interest of the animal, the Mississippi State University Institutional Care and Animal Use Committee determined that official animal use approval was not needed. Informed consent for enrolment, including a signed consent form, was obtained from all dog owners.

### Statistical methods

The initial project design allowed for inclusion of up to 50 dogs, over a period of up to 2 years, with initial large patient numbers based on uncertainty regarding rates of attrition and likely response rates for Phase One of the project.

Data were then analysed after completion of the cases enrolled in the first full year of the project. The proportion of dogs which remained enrolled in the study (that is, not lost to attrition), that successfully completed either Phase One with complete resolution of diarrhoea, or a combination of both Phase One and Phase Two with complete resolution of diarrhoea, was compared to a predetermined minimum success rate of 50% using an exact binomial test (PROC FREQ in SAS for Windows v9.4, SAS Institute, Inc., Cary, NC, USA). Clopper‐Pearson exact confidence intervals were calculated assuming an alpha level of 0.05.

## Results

Twenty‐two dogs were enrolled in the study. Median patient age was 2 years old, with ages ranging from 8 months to 12 years. Eight patients were female (all neutered) and 14 patients were male (five intact, nine neutered). Enrolled dog breeds included four German Shepherds, three mixed breed dogs, two Chihuahuas, two Dobermans and one Beagle, Belgian Malinois, Bluetick Coonhound, Boston Terrier, Cocker Spaniel, Dachshund, English Bulldog, Labrador Retriever, Pomeranian, Weimaraner and Yorkshire Terrier. Eleven dogs had predominantly large bowel diarrhoea, eight dogs had predominantly small bowel diarrhoea and three dogs had a mix of small and large bowel diarrhoea.

Thirteen dogs had complete resolution of diarrhoea during the Phase One of the study, with no relapse over the following month and were therefore considered to be treatment successes. Three dogs were lost to the study, early in Phase One, because of a failure of owners to attend revisit appointments or to respond to communication attempts. One dog died, during Phase One of the study, due to acute heartworm caval syndrome. Five dogs failed Phase One of the study (persistent diarrhoea despite therapeutic interventions) and were enrolled in Phase Two of the study.

Final diagnosis was difficult to establish for most dogs in which diarrhoea resolved during Phase One of the study, because the key focus of cost‐effective management protocols was to attain resolution of diarrhoea and in many instances this outcome could be achieved without establishment of a definitive diagnosis. Therapeutic trials were conducted in a step‐wise approach (Allenspach *et al*. 2016; Volkmann *et al*. 2017) and, once a response to therapy was observed, typically no further diagnostic tests or therapeutic trials were conducted. But, of the 13 dogs that responded, five responded to a diet trial alone [four to a commercial single protein and carbohydrate source highly digestible diet (Hill's Prescription Diet d/d, canned and dry formulations, Hill's Pet Nutrition, Topeka, KS, USA), one to a commercial high fibre diet (Hills Prescription Diet r/d, canned and dry formulations, Hill's Pet Nutrition)], one responded to a combined diet (commercial single protein and carbohydrate source highly digestible diet) and anthelmintic (fenbendazole) trial, two responded to a combined diet (commercial single protein and carbohydrate source highly digestible diet) and fibre (psyllium) trial, one responded to a combined diet (commercial single protein and carbohydrate source highly digestible diet), fibre (psyllium) and probiotic trial, one responded to an anthelmintic (fenbendazole) trial, one responded to a combined anthelmintic (fenbendazole) and antibiotic (metronidazole) trial, one responded to an antibiotic (metronidazole) trial and one responded to a combined diet (commercial single protein and carbohydrate source highly digestible diet), anthelmintic (fenbendazole), antibiotic (tylosin) and probiotic trial.

Of the five dogs that failed to respond to Phase One and moved on to Phase Two, evaluation of endoscopically guided gastrointestinal mucosal pinch biopsies was required as a key component of the work‐up in all dogs in order to establish a final diagnosis. Four of the five dogs eventually responded to appropriate therapy based on the established final diagnosis. Final diagnoses in the four dogs that responded to therapy were moderate to focally severe mixed lymphocytic plasmacytic and eosinophilic inflammatory bowel disease (IBD) of the small intestine and mild to moderate lymphocytic plasmacytic colitis in one dog (sustained response to a commercial single protein and carbohydrate source highly digestible diet, a probiotic, tapering doses of oral prednisolone and ongoing therapy with oral cyclosporine), mild mixed lymphocytic plasmacytic and eosinophilic IBD of the small intestine in one dog [initial response to a commercial hydrolysed diet (Hills Prescription Diet z/d, canned and dry formulations, Hill's Pet Nutrition), oral prednisolone and oral cyclosporine, with eventual long‐term remission on a hydrolysed diet alone], moderate mixed lymphocytic plasmacytic and suppurative IBD of the small intestine in one dog (initial response to a commercial hydrolysed diet, oral metronidazole, oral prednisolone and a probiotic, with eventual long‐term remission on a probiotic alone) and equivocal or mild IBD of the small intestine on histopathology but strong positive fluorescence in situ hybridization staining for adherent and invasive bacteria on the same mucosal biopsy specimens in one dog, suggestive of a bacteria‐associated enteritis (failed to respond to trials with multiple antibiotics and dietary modification, but eventual response to a faecal transplant, cobalamin and ongoing therapy with oral prednisolone and cyclosporine). One dog from this group was prematurely moved to Phase Two of the project after 3 weeks, because of significant weight loss, rather than completing the full course of Phase One diagnostic and therapeutic trials. Final diagnosis in the one dog that failed to respond to therapy was moderate mixed lymphocytic plasmacytic and suppurative IBD of the small intestine and mild to moderate lymphocytic plasmacytic colitis, with concurrent severe (suspected hereditary) immunoglobulin G deficiency. This dog was prematurely moved to Phase Two of the project after 2 weeks, because of marked hypoalbuminemia, rather than completing Phase One. The diarrhoea initially responded to a commercial single protein and carbohydrate source highly digestible diet and tapering doses of oral prednisolone and cyclosporine, but relapsed during episodes of poor owner dietary compliance. The dog also had multiple episodes of antibiotic‐responsive pneumonia suspected to be due to the combination of immunoglobulin G deficiency and immunosuppressive therapy and was eventually euthanised 1 year after initial diagnosis.

The study was concluded after 1 year of data collection because of the high rates of resolution of diarrhoea in both phases of the study. Thirteen out of 18 dogs completed Phase One of the study with complete resolution of diarrhoea, providing a success rate of 72.2% (confidence intervals 46.5–90.3%). The observed success rate was not different (*P* = 0.096) than a predetermined approximate minimum success rate of 50%. Five out of 18 dogs did not have complete resolution of diarrhoea during Phase One of the study and moved on to Phase Two, consistent with a Phase One failure rate of 27.8% (confidence intervals 9.7–53.5%). Seventeen out of 18 dogs completed a combination of Phase One and, if necessary, Phase Two, of the study with complete resolution of diarrhoea, providing an overall success rate of 94.4% (confidence intervals 72.7–99.9%). The observed success rate was different (*P* = 0.0001) than a predetermined approximate minimum success rate of 50%.

Over the course of the study, the authors developed two broad template protocols, one for small bowel diarrhoea and one for large bowel diarrhoea (Tables 1 and 2).

**Table 1:** Small bowel diarrhoea protocol

Week 1	Faecal float If faecal float negative: Fenbendazole 50 mg/kg orally daily for 5 days (Barr *et al*. 1994; Plumb 2018) Commence limited antigen or hydrolysed diet dietary trial (Zoran 2003; Mandigers *et al*. 2010; Westermarck 2016) If faecal float positive: Roundworm or hookworm Fenbendazole [50 mg/kg (23 mg/lb) orally daily], one 3‐day course (Plumb 2018) Whipworm Fenbendazole three 3‐day course [50 mg/kg (23 mg/lb) orally daily], immediately, then again at 3 weeks and 3 months (Plumb 2018) Coccidia Sulphadimethoxine 55 mg/kg (25 mg/lb) 1st day, then 27.5 mg/kg (12.5 mg/lb) daily for 9 days (Plumb 2018) Spirometra Praziquantel at label dosage (Plumb 2018) Plus appropriate environmental control as needed Then repeat float 10–14 days later
Week 2 (if diarrhoea persists)	Bloodwork CBC, serum biochemical profile chemistry, including electrolytes Triggers for switching to thorough investigation: Albumin <1.8 mg/dL; or significant dehydration, weight loss, or anorexia Continue current diet trial Commence probiotic (Sauter *et al*. 2006; Rossi *et al*. 2014; Schmitz *et al*. 2015a,b) Commence antibiotic trial (Kilpinen *et al*. 2015) Tylosin 25 mg/kg (11.4 mg/lb) orally once daily for 7 days (Kilpinen *et al*. 2015)
Week 3 (if diarrhoea persists)	Perform send‐out gastrointestinal panel Trypsin‐like immunoreactivity/folate/cobalamin and cortisol Administer cobalamin (Toresson *et al*. 2016) Empirically while awaiting results or if GI panel not affordable Continue probiotic, antibiotic and current diet trials
Weeks 4–6 (if diarrhoea persists)	Rectal scrape In areas endemic for organisms such as *Pythium* or *Histoplasma* (Wilson *et al*. 2018) Continue current diet trial Change probiotic (Sauter *et al*. 2006; Rossi *et al*. 2014; Schmitz *et al*. 2015a,b) Change antibiotic Metronidazole 10 mg/kg (4.5 mg/lb) orally 2–3 times daily for 7 days (Hall 2011) If not improved by end of week 6, commence specialist‐level investigation
Selected scenarios	Irish Setter: Gluten free diet trial (Hall & Batt 1992) Yorkshire Terrier: Low fat diet trial (Okanishi *et al*. 2014; Rudinsky *et al*. 2017) Norwegian Lundehund: Consider protein‐losing enteropathy early (Berghoff *et al*. 2007) German Shepherd: Consider trypsin‐like immunoreactivity and antibiotic‐responsive diarrhoea early (Hall 2011) Standard Poodle, Nova Scotia Duck Tolling Retriever: Consider cortisol early (Hughes *et al*. 2007; Friedenberg *et al*. 2017)

Protocol is not intended to be rigidly adhered to and flexible modifications are recommended based on individual patient circumstances.

**Table 2:** Large bowel diarrhoea protocol

Weeks 1–2	Faecal float Faecal Gram stain Faecal wet mount Faecal *Giardia* SNAP test If faecal tests negative: Fenbendazole 50 mg/kg (22.7 mg/lb) orally daily for 5 days (Barr *et al*. 1994; Plumb 2018) Commence high fibre diet dietary trial (Lecoindre & Gaschen 2011) If faecal float positive: Roundworm or hookworm Fenbendazole [50 mg/kg (22.7 mg/lb) orally daily], one 3‐day course (Plumb 2018) Whipworm Fenbendazole three 3‐day course [50 mg/kg (22.7 mg/lb) orally daily], immediately, then again at 3 weeks and 3 months (Plumb 2018) Coccidia Sulphadimethoxine 55 mg/kg (25 mg/lb) 1st day, then 27.5 mg/kg (12.5 mg/lb) daily for 9 days (Plumb 2018) Spirometra Praziquantel at label dosage (Plumb 2018) Plus appropriate environmental control as needed Then repeat float 10–14 days later If faecal Gram stain positive: *Clostridium* (over 15 organisms/high power field), give metronidazole 10–15 mg/kg (5.5–6.8 mg/lb) orally twice daily for 5 days (Plumb 2018) If wet mount or *Giardia* SNAP positive: Give metronidazole 30 mg/kg (13.6 mg/lb) orally twice daily for 3–5 days (Reddy *et al*. 1992)
Week 3 (if diarrhoea persists)	Commence limited antigen dietary trial (Zoran 2003; Mandigers *et al*. 2010; Westermarck 2016) Commence concurrent fibre (Lecoindre & Gaschen 2011) Psyllium; ½ tablespoon daily toy breed, 1 tablespoon daily small breed, 2 tablespoons daily medium breed, 3 tablespoons daily large breed Commence probiotic trial (Sauter *et al*. 2006; Rossi *et al*. 2014; Schmitz *et al*. 2015a,b) If faecal tests positive for organisms Weeks 1–2, and treated, repeat test: Consider alternate therapies if still positive
Week 4 (if diarrhoea persists)	Continue current diet and concurrent fibre trials Change probiotic (Sauter *et al*. 2006; Rossi *et al*. 2014; Schmitz *et al*. 2015a,b)
Week 5 (if diarrhoea persists)	Continue current diet and concurrent fibre trials Commence antibiotic trial (Kilpinen *et al*. 2015) Tylosin 25 mg/kg (11.4 mg/lb) orally once daily for 7–14 days (Kilpinen *et al*. 2015)
Week 6 (if diarrhoea persists)	Continue current diet and concurrent fibre trials Rectal scrape (Wilson *et al*. 2018) Faecal transplant (Chaitman *et al*. 2016; Bottero *et al*. 2017) If not improved by end of week 6, commence specialist‐level investigation
Selected scenarios	Young Boxers, English bulldogs, French bulldogs that have failed initial standard large bowel diarrhoea protocol: Enrofloxacin 5–10 mg/kg (2.3–4,6 mg/lb) once daily 6 weeks (Hostutler *et al*. 2004; Craven *et al*. 2011)

Protocol is not intended to be rigidly adhered to and flexible modifications are recommended based on individual patient circumstances.

## Discussion

Thorough investigation of chronic diarrhoea at a specialist level can involve a comprehensive diagnostic approach that typically includes a CBC, serum biochemical profile, faecal flotation, abdominal ultrasound and upper and/or lower gastrointestinal endoscopy with intestinal mucosal biopsy, frequently accompanied by supportive tests such as a range of specialised assays for faecal microorganisms and measurement of serum folate, cobalamin and trypsin‐like immunoreactivity (Leib 2000; Allenspach *et al*. 2007; Fogle & Bissett 2007; Volkmann *et al*. 2017). Standard and thorough investigations of this nature are, however, typically relatively expensive and may be beyond the economic means of many pet owners. In these circumstances, veterinarians must consider alternative, cost‐effective approaches to the management of chronic diarrhoea and, if such an approach is utilised, a key factor that must be considered as the likelihood of success in the absence of a standard thorough investigation.

The authors of this study are based at a veterinary teaching hospital that services a number of the poorest states in the United States in terms of median household income. Commonly, clients of the veterinary teaching hospital and clients of the regional veterinarians that consult with the teaching hospital, decline recommended standard investigations for primarily financial reasons. In these circumstances, ‘compromise’ approaches that are centred primarily on simple diagnostic tests and cost‐effective therapeutic trials are commonly recommended. The authors’ experience has been that for some conditions, such as chronic diarrhoea in dogs, treatment success rates using cost‐effective approaches do not appear to be markedly lower than success rates utilising accepted ‘standard of care’ approaches. Specifically, for chronic diarrhoea, the authors hypothesised that cost‐effective treatment success rates would exceed 50%. Our study demonstrated that over 70% of the dogs (confidence intervals approximately 45–90%) that remained enrolled in our study experienced sustained resolution of diarrhoea using a targeted budget‐restricted approach that costs no more than $300 for any dog.

High success rates with therapeutic trials using a predominantly empirical approach are not unexpected. A number of previous studies of chronic diarrhoea in dogs, that typically utilised much more extensive diagnostic approaches than those used in this current study, demonstrated a high prevalence of conditions that would be amenable to consideration of a simple therapeutic trial *in lieu* of a complete investigation (Leib 2000; Lecoindre & Gaschen 2011; Allenspach *et al*. 2016; Volkmann *et al*. 2017). Lieb, for example, reported a high success rate utilising a highly digestible diet and soluble fibre (psyllium) in dogs with chronic idiopathic large bowel diarrhoea (Leib 2000). More recently, Volkmann and others reported a high prevalence of food‐responsive enteropathies (47% of the affected dogs, with patients typically responding to an elimination diet) and antibiotic‐responsive enteropathies (8% of the affected dogs, with patients typically responding to metronidazole) in a large series of dogs with chronic diarrhoea (Volkmann *et al*. 2017) and Allenspach and others, in another large series of dogs with chronic enteropathies, reported a 64% prevalence of food‐responsive diarrhoea and a 16% prevalence of antibiotic‐responsive diarrhoea (Allenspach *et al*. 2016).

The intention of this study was not to develop single, inflexible cost‐effective management protocols to be applied to all patients. Standard consultation fees were included in the fee structure of all of the cases enrolled in this study and, as such, the owners should expect to benefit from the full expertise of the attending veterinarian. This expertise includes the ‘art’ of veterinary medicine: that is, individualising management approaches based on patient factors (signalment, history, physical examination and the results of simple diagnostic tests) and on owner factors (including budget expectations, geographic and travel limitations and likelihood of compliance). Our study, therefore, did not demonstrate the effectiveness of any single protocol for the management of chronic diarrhoea and, instead, demonstrated that a careful, evidence‐based, structured and individualised approach, with an emphasis on cost‐effectiveness, has a high likelihood of success. Case‐by‐case factors, such as current cost and availability, were considered when, for example, determining which probiotic, fibre source or diet to initially utilise. Nevertheless, before and during the study, the authors developed several broad template protocols that served as a starting point for individualising therapy in specific patients. Such protocols would be expected to evolve over time, as new diagnostic and therapeutic approaches are published.

One limitation of our study is that it is not possible to distinguish spontaneous remission of diarrhoea from resolution that is a direct result of therapeutic intervention. A second, untreated control group would have been required in order to determine the rate of spontaneous remission but, in the authors’ opinion, it would not have been ethical to maintain an untreated group of dogs with chronic diarrhoea. The authors chose to define ‘chronic’ diarrhoea as diarrhoea that has persisted for at least 2 weeks with the expectation that, with diarrhoea that had been present for this duration of time, the rate of spontaneous remission would be significantly lower than it would be for episodes of acute diarrhoea. Some authors define chronic diarrhoea in dogs as diarrhoea that has been present for at least 3 or 4 weeks (Leib 2000; Volkmann *et al*. 2017), but the authors did not wish to withhold treatment of diarrhoea for this extended time.

One unexpected finding of our study was that maintaining owner compliance with diagnostic and therapeutic recommendations proved to be highly challenging. We had mistakenly assumed that, as the veterinary fees to the owners of pets enrolled in the study were entirely subsidised, owners would be incentivised and have high rates of compliance. However, a number of patients were lost to the study due to loss of contact with owners and, for many of the owners with dogs that successfully completed the study, compliance was dependent on extensive follow‐up and repeated reminders. Multiple factors beyond simple health care fees can contribute to a lack of compliance in human low income groups that suffer from chronic disease, including lack of trust in health care systems, an inability to be absent from employment, a lack of available transportation, a lack of means of communication such as telephone lines, cell phones, computers and internet connectivity and a high rate of geographic instability (Wamala *et al*. 2007; Zeber *et al*. 2013; Stankunas *et al*. 2014). Based on our experience with this study, we suspect that similar factors can affect compliance within the population of low income pet owners. Since budget‐limited treatment approaches, particularly therapeutic trials, are largely dependent on clear and regular communication with clients, the authors consider that development of effective communication strategies, such as regularly scheduled text reminders, will be essential to the success of such approaches.

Another unexpected finding of our study was that many collaborating veterinarians in general practice were not familiar with and therefore not comfortable performing what the authors considered to be simple and economical diagnostic tests and therapeutic strategies such as, for example, faecal Gram stains and rectal scrape cytology. In order to facilitate collaborating veterinarian performance of these cost‐effective techniques, the authors developed a series of instructional hand‐outs specifically outlining simple and useful diagnostic and therapeutic methodologies and augmented these with narrated instructional videos available through a freely accessible web link (http://www.cvm.msstate.edu/animal-health-center/chronic-diarrhea-project). Most North American veterinarians are graduates of veterinary teaching hospitals that have a significant proportion of specialist and referral‐based clinical teaching rotations and, as a result, students may not necessarily be adequately exposed, prior to graduation, to the full range of simple and cost‐effective choices and techniques that are feasible in general practice (Roder & May 2017). One observation that arose from the authors’ experience with this study was the need to provide a greater emphasis on the teaching of simple, affordable techniques to both veterinary students and practising veterinarians.

The treatment success rates observed in our study may not necessarily be extrapolated to other geographic areas, as the prevalence of conditions that cause chronic diarrhoea may vary with location. The prevalences of purely small bowel diarrhoea, large bowel diarrhoea and mixed small/large bowel diarrhoea in our study, for example, were 36%, 50% and 14%, respectively, whereas a large study of dogs with chronic diarrhoea in Germany, prevalences of small bowel, large bowel and mixed diarrhoea were 33%, 28% and 39%, respectively, suggesting the potential for a different distribution of underlying disease (Volkmann *et al*. 2017). Nevertheless, both studies reported a high success rate with initial treatments based predominantly on therapeutic trials. Protocol modifications may, however, be indicated based on disease prevalence in specific geographic regions. In Europe, for example, where mixed small/large bowel diarrhoea is more prevalent (Volkmann *et al*. 2017), measurement of serum folate and cobalamin may be indicated earlier in the investigative process, particularly when small intestinal involvement is suspected in dogs in which signs of large bowel diarrhoea predominate.

While all of the dogs in our study that responded to a budget‐limited affordable protocol did so for a total cost of under $300 (up to and including the first month of complete resolution of diarrhoea), geographic variations in the cost of veterinary services would most likely lead to variations in what would be considered an ‘affordable’ protocol in different areas. For example, based primarily on the costs of testing serum cobalamin levels within our region, the protocol developed for small bowel diarrhoea in our study delayed testing serum cobalamin levels until the third week of the protocol. In areas where costs of cobalamin testing are lower, earlier testing may be indicated. Early empirical therapy with cobalamin could also be considered. Although hypocobalaminaemia is usually secondary to a primary gastrointestinal disease, failure to normalise serum cobalamin may impair the response to other treatment trials.

## Conclusion

In summary, our study confirmed that many dogs with chronic diarrhoea could be successfully managed using tightly budget‐restricted evidence‐based and individualised management protocols. A step‐wise approach to dogs with chronic diarrhoea, with an emphasis on affordable therapeutic trials and limited diagnostic testing, is feasible in selected canine patients with owners of limited financial means. More extensive diagnostic and therapeutic interventions, however, will still be indicated in some patients, particularly those dogs that are systemically unwell, that fail to respond to therapeutic trials or that do not have financial restrictions that limit their management approach. Based on the results of our study, an initial budget‐restricted management protocol centred primarily on therapeutic trials, followed by aggressive diagnostic investigation limited only to those dogs that fail to respond to initial therapy with a targeted treatment focused on a final diagnosis, has a high likelihood of attaining successful resolution of diarrhoea, with an overall success rate of 94% (confidence intervals approximately 70–100%). Our study demonstrates that a complete, thorough investigation of chronic diarrhoea can be deferred while simple affordable diagnostics and therapeutic trials are conducted in stable patients and, in many instances, an extensive management approach will not be needed.

## Source of funding

Funding support for this project was provided by a grant from the Stanton Foundation. The funding source did not have any involvement in the study design, data analysis and interpretation or writing and publication of the manuscript.

## Conflicts of interest

The authors declare that they have no conflicts of interest.

## Ethics statement

The authors confirm that the ethical policies of the journal, as noted on the journal's author guidelines page, have been adhered to and the appropriate ethical review committee approval has been received.

## Contributions

All of the four authors were involved in every aspect of the study, and contributed equally to study development, completion and manuscript preparation.
